# Efficacy of Technology-Based Interventions on the Reduction of Loneliness: Systematic Review and Meta-Analysis

**DOI:** 10.2196/80059

**Published:** 2026-05-08

**Authors:** Zdenek Meier, Marie Buchtova, Jan Sandora, Lukas Novak, Jakub Helvich, Ondrej Buchta, Jana Furstova, Klara Malinakova, Peter Tavel

**Affiliations:** 1 Olomouc University Social Health Institute, Palacký University Olomouc, Univerzitni 244/22, Olomouc, 771 11, Czech Republic, 420 585637394

**Keywords:** loneliness, technology, intervention, meta-analysis, systematic review

## Abstract

**Background:**

Loneliness is a widespread public health concern linked to increased risks of health problems. As populations age, the demand for effective interventions to mitigate loneliness continues to grow.

**Objective:**

This meta-analysis aimed to examine the effectiveness of technology-based interventions in reducing loneliness.

**Methods:**

A systematic literature search was conducted in Web of Science, PsycInfo, PubMed, Scopus, Google Scholar, Embase, and the Cochrane Library in August 2024. We included randomized controlled trials that examined the effectiveness of technology-based interventions compared with any control group in reducing loneliness across all age groups. Nonrandomized studies, qualitative research, and studies lacking sufficient statistical data for effect size calculation were excluded. After screening 1089 records, 7 studies involving 580 participants met the inclusion criteria. Data were extracted by 3 independent reviewers, with discrepancies resolved by a fourth reviewer. The risk of bias was assessed using the Cochrane risk-of-bias tool. A random-effects model was used to synthesize effect estimates, with standardized mean differences as the primary effect size metric. Heterogeneity was assessed using the Q statistic and *I*² index, and a prediction interval was calculated to estimate the expected range of true effects.

**Results:**

We found a small and statistically nonsignificant overall effect of technology-based interventions on loneliness (pooled standardized mean difference=–0.21, 95% CI –0.59 to 0.17; 95% prediction interval –1.14 to 0.63). Substantial between-study variability was present (*I*²=57%; τ²=0.08; *τ*=0.28), and the prediction interval indicated that true effects in future studies may range from substantial reductions to moderate increases in loneliness. Differences between intervention types could not be examined due to the limited number of eligible studies. The Egger test showed significant funnel plot asymmetry (*t*_5_=–5.47; *P*=.003). However, since the trim-and-fill method identified no missing studies, the asymmetry is unlikely to be fully explained by publication bias.

**Conclusions:**

Unlike previous reviews, which focused primarily on older populations or included nonrandomized studies, this meta-analysis provides a rigorous synthesis of only randomized controlled trials across the lifespan. Our findings do not provide evidence for a reliable reduction in loneliness following technology-based interventions. Moderate heterogeneity indicates that effects differ across studies, and the limited number of eligible trials prevented the analysis of potential moderators. By incorporating robust prediction intervals and a broader age demographic, this study offers a more comprehensive view of the variability of intervention outcomes compared with existing literature. The wide prediction interval suggests that intervention effects may vary across settings. In practice, technology-based approaches should be viewed as supportive tools that require careful tailoring rather than universal solutions to loneliness.

## Introduction

### Negative Impacts of Loneliness

Loneliness is a growing public health challenge linked to a wide range of adverse mental and physical health outcomes. It has gained considerable attention in recent years due to its significant impact on both mental and somatic health. It is a major risk factor for various physical health problems, influencing biological responses to stress [1,2], glucose regulation [3], cardiovascular risk [4,5], and overall health behaviors [6]. Furthermore, loneliness is a critical risk factor for mental health, as individuals experiencing loneliness exhibit higher levels of psychological distress, depression, and poorer subjective health assessments [7]. It is associated with an increased prevalence of paranoid symptoms [8], depression [9,10], anxiety [10,11], and psychotic symptoms [8], as well as lower self-esteem and self-evaluation [12,13]. A study by Liang et al [14] identified loneliness as a risk factor for more than two-thirds of the analyzed diseases and for 13 out of 14 disease categories, particularly mental and behavioral disorders, infectious diseases, and conditions affecting the nervous, respiratory, and endocrine systems [14]. Lastly, loneliness is associated with an increased risk of mortality [15,16].

Loneliness encompasses both adaptive and maladaptive aspects. In the cognitive model, short-term loneliness can function as a social mechanism, prompting individuals to strengthen interpersonal bonds and seek social connections [17]. This adaptive process can be considered an evolutionary response to the fundamental human need for belonging [18]. However, chronic loneliness leads to negative consequences affecting both physical and mental health [17,19,20]. The model of loneliness as an adaptive mechanism with maladaptive consequences is essential not only for understanding its biological function but also for recognizing the risks associated with its long-term presence in an individual’s life. Loneliness can be helpful in the short term, but chronic loneliness leads to poorer health and reduced life satisfaction [21].

Loneliness has widespread societal impacts, including economic consequences such as increased health care costs and reduced work productivity [22]. Effective solutions to loneliness lie in future research and interventions aimed at preventing its occurrence and mitigating its negative effects on both individuals and society [23-25]. As a significant public health issue, loneliness requires a multidisciplinary approach involving collaboration among experts in health care, psychology, and social sciences [24,26,27]. Key strategies for addressing loneliness are particularly found in social work and social policies, which can help reduce risk factors and promote social inclusion [26,28].

### Definition

From a multidimensional perspective, loneliness can be defined as a significant psychological and social phenomenon, primarily conceptualized in the literature as the subjective perception of a discrepancy between expected and actual social relationships [29]. This discrepancy is not necessarily objectively measurable by the number of social interactions but rather by the qualitative evaluation of one’s relationships [30]. Moreover, loneliness is a complex phenomenon encompassing both affective and cognitive components. The affective component relates to the emotional distress associated with feelings of isolation, whereas the cognitive component reflects the perceived inadequacy of social relationships [21].

Weiss [31] distinguished between social loneliness, which is related to a lack of social networks and a sense of social exclusion, and emotional loneliness, which is associated with the absence of close and meaningful relationships [31]. In addition to these, another critical dimension is existential loneliness, characterized by a profound sense of isolation linked to questions of life’s meaning, human finiteness, and the subjective incommunicability of individual experience [32].

Based on these observations, loneliness can be explored according to its 3 dimensions: intimate loneliness, which pertains to the lack of a deep relationship with a close person; relational loneliness, which arises from the absence of quality friendships or family connections; and collective loneliness, which stems from a missing sense of belonging to a group or a broader community [33-35]. The concept of collective loneliness remains a subject of debate and requires further investigation and clarification [36].

When examining the relationship between loneliness and the quality and quantity of social relationships, particularly among older adults, it is essential to consider not only quantitative aspects, such as the number of friends and frequency of interactions, but also the perception of social support and emotional closeness, which determine the quality of relationships [30].

### The Prevalence of Loneliness

Loneliness is highly prevalent across the lifespan and in diverse sociodemographic groups [7-9]. Population-based studies and meta-analyses indicate that roughly 1 quarter to one third of adolescents and older adults report at least moderate levels of loneliness, with substantial variation between countries and regions [37-48]. Although some longitudinal studies suggest a U-shaped or more complex trajectory, with elevated loneliness particularly during adolescence and in later life [37,49-51], these patterns appear to be driven by life transitions and changes in social roles rather than age per se [40,41,49]. Rates of loneliness increased further during the COVID-19 pandemic, when large-scale public health measures such as social distancing and lockdowns disrupted everyday social contact [52-56].

Chronic loneliness is especially concerning; estimates suggest that about 1 in 5 older adults experience persistent loneliness, which is most strongly associated with adverse physical and mental health outcomes [47,57]. Against this backdrop, there is an urgent need for scalable, evidence-based interventions that can be implemented across different age groups and settings.

### Technology-Based Interventions for Loneliness

In recent years, digital technology has emerged as a promising avenue for delivering loneliness interventions. Technology-based interventions are approaches that use digital or communication technologies as the primary mode of delivery—for example, web-based programs, mobile apps, videoconferencing platforms, or socially assistive robots designed to enhance social connection or provide psychosocial support [58]. These interventions can extend the reach of traditional face-to-face programs, particularly for individuals who are homebound, geographically isolated, or affected by situations such as the COVID-19 pandemic, where physical distancing measures increased both loneliness and the need for remote support [58,59]. By enabling people to connect with peers, family members, or professional services via digital means, they offer a potentially scalable way to address loneliness [59,60]. However, the existing evidence base remains limited and heterogeneous, with many small trials of variable quality and relatively few rigorous randomized controlled studies [58].

Existing systematic reviews of loneliness interventions have often focused on traditional face-to-face programs or on specific subgroups such as older adults, and several recent reviews of digital interventions report small or nonsignificant effects, substantial heterogeneity, and methodological limitations. As new trials using diverse technologies and age groups have accumulated, an updated and methodologically rigorous synthesis is needed to clarify under what conditions technology-based interventions can meaningfully reduce loneliness.

Thus, this study aims to conduct a systematic review and meta-analysis to evaluate the effectiveness of technology-based interventions compared with control conditions in reducing loneliness among individuals across the lifespan. We focus on interventions in which digital or communication technologies constitute the main delivery channel (eg, mobile apps, web-based self-help programs, videoconferencing, socially assistive robots). Specifically, we address the following research questions:

1. To what extent do technology-based interventions reduce loneliness compared with control conditions?
2. Do the effect sizes differ between distinct types of technology-based interventions (eg, app-based psychological programs, socially interactive technologies, videoconferencing-based approaches)?
3. Are intervention or sample characteristics such as participant age, intervention duration, and follow-up period associated with differences in effectiveness?

Clarifying these questions is particularly important in the context of population aging and growing shortages in health and social care workforces, where scalable digital solutions may help to complement, but not replace, traditional forms of support.

## Methods

### Research Design

The protocol for this study was registered with the PROSPERO (International Prospective Register of Systematic Reviews) under the registration number CRD42024505117 [61]. The systematic review and meta-analysis were conducted and reported in accordance with the PRISMA (Preferred Reporting Items for Systematic Reviews and Meta-Analyses) statement [62,63], using the PRISMA 2020 expanded checklist to ensure comprehensive reporting. The literature search was carried out following the PRISMA-S extension for reporting search strategies [64], and the abstract was structured according to the PRISMA 2020 for Abstracts guidelines. Fully completed versions of all relevant PRISMA checklists are provided in Checklist 1.

### Eligibility Criteria

To ensure methodological rigor, this meta-analysis included studies meeting the following criteria: (1) no restrictions on publication language; (2) no restrictions regarding the age of participants; (3) empirical studies using a randomized controlled trial (RCT) design; (4) use of a technology-based intervention compared with a control group; and (5) reporting of loneliness as a measured outcome (either as a primary or secondary end point), with available loneliness levels before and after the intervention or sufficient data to calculate the effect size. Furthermore, no restrictions were placed on the study setting (eg, community-dwelling or long-term care facilities) or the minimum duration of the intervention. Regarding the definition of loneliness, studies were eligible if they operationalized the construct consistent with the conceptual framework described in the *Introduction* section, assessing subjective feelings of loneliness or social isolation using quantitative measures. Studies that did not meet all of these criteria, including nonempirical studies, non-RCT designs, interventions unrelated to technology, and those lacking relevant outcome measures, were excluded.

### Search Strategy

The literature search for this meta-analysis was conducted in October 2023, with a final update performed on August 21, 2024. The following databases were systematically searched from their respective inception dates: Web of Science (Clarivate), PsycInfo (EBSCOhost), PubMed (NCBI), Scopus (Elsevier), Google Scholar, Embase (Elsevier), and the Cochrane Library (Wiley). To identify relevant studies, search terms were organized into four thematic groups: (1) loneliness (eg, “loneliness,” “perceived social isolation”); (2) intervention (eg, “intervention,” “treatment”); (3) technology (eg, “technology,” “computer,” “mobile,” “app*”); and (4) study design (eg, “Randomized Controlled Trial,” “RCT”). Searches were conducted in the fields of Title, Abstract, and Keywords, except in Google Scholar, which did not support specific operators for limiting searches to these fields. Boolean operators and truncation symbols were used to ensure a comprehensive and systematic identification of studies relevant to technology-based interventions for reducing loneliness. The exact search syntaxes applied to each database are detailed in Multimedia Appendix 1. In addition to database searches, further studies were identified through citation searching in the reference lists of relevant review articles on the topic (see details below).

### Selection Process

All retrieved citations were imported into Zotero reference management software [65]. Duplicate records were identified and removed using the software’s automated functions, followed by manual verification. Titles and abstracts underwent an initial screening to determine relevance. First, records that did not report published results of primary empirical research were excluded. This included books, study protocols, preprints, review articles (including meta-analyses), theoretical studies, conference contributions, master’s theses, commentaries, editorials, letters to the editor, and studies with inaccessible full texts. Initially, we also intended to exclude dissertation theses. However, we decided to retain them in the search, as we consider the dissertation review process to be sufficiently rigorous. Second, studies that were thematically irrelevant were excluded. This applied to records that did not focus on loneliness (eg, loneliness was not measured as an outcome) or did not examine the impact of technology-based approaches (eg, the intervention was not technology-driven, or the study did not compare nontechnology vs technology-based methods). Third, studies that did not follow an RCT design were excluded. This included, for example, studies assessing the feasibility of RCTs, those using qualitative designs, or other nonexperimental approaches.

Thematically relevant reviews were used for further citation searching to help identify additional empirical studies not captured in the initial database search. The full texts of studies were reviewed based on predefined inclusion and exclusion criteria. The selection process was conducted by 3 reviewers. Each record and report was screened independently by at least 2 reviewers. Any discrepancies were resolved through consultation with a fourth reviewer. All stages of the study selection process were conducted manually; no automation tools or machine learning classifiers were used.

There were minor deviations from the original PROSPERO protocol. First, the literature search was updated in August 2024 to include the most recent evidence, extending the search period beyond the date initially planned. Second, while the protocol mentioned a dedicated search for gray literature, the final strategy relied on comprehensive database searches and citation tracking of relevant reviews. Finally, the GRADE (Grading of Recommendations Assessment, Development, and Evaluation) approach and the calculation of prediction intervals were added during the analysis stage to provide a more robust assessment of evidence certainty and effect distribution.

### Data Collection Process

A predefined data extraction form was used to collect the following information: (author, year, country), participant demographics (sample size, characteristics, age range, mean age, and gender distribution), baseline loneliness levels, measurement instruments, and intervention details (type of intervention and control, duration, and the timing of postintervention assessment). For all included studies, loneliness data were extracted using the specific quantitative measure provided by the authors. In cases where studies reported results from multiple postintervention time points, data from the assessment conducted immediately following the completion of the intervention were extracted to ensure consistency. Data extraction was conducted by 3 reviewers, with each study independently coded by 2 reviewers. Any discrepancies were resolved by involving a fourth reviewer. The process was performed manually, and no automation tools or machine learning applications were used. All relevant data were obtained through a comprehensive review of the text, tables, and figures of each report; no data extraction software for figures was required. As all included studies were published in English, no formal translation process was necessary. Only studies reporting sufficient statistical information to compute pre-post effect sizes were included. Thus, according to this criterion, 8 studies were excluded. We chose not to contact study authors for missing data because of practical constraints and the likelihood of delayed or incomplete responses. To ensure transparency and replicability, we decided that only studies with fully reported data necessary for effect size computation (including pre-post correlations) were included. This approach allows the meta-analysis to rely only on verifiable and publicly available information.

### Data Synthesis

A random-effects model was used to synthesize effect estimates across studies, accounting for between-study heterogeneity. Effect sizes and their sampling variances were calculated as standardized mean differences (SMDs), specifically the difference between standardized mean changes in the intervention and control groups. This metric was chosen to accurately account for the pre-post study design and to enable the comparison of results from studies using different loneliness measurement scales [66]. The magnitude of the pooled effect size was interpreted according to Cohen [67] conventional criteria: 0.2 (small), 0.5 (medium), and 0.8 (large). All computations were performed in R using the metafor package. The summary effect size and its 95% CI were computed to estimate the overall intervention effect. A random-effects model was fitted using restricted maximum likelihood, and the Hartung-Knapp-Sidik-Jonkman [68] adjustment was applied to obtain robust confidence intervals given the small number of studies (k=7). Additionally, a prediction interval was calculated using the bootstrap confidence distribution approach proposed by Nagashima et al [69] to define the expected range of true effects in comparable populations and future studies, as the prediction interval quantifies the range within which the true effect sizes are expected to fall, providing a clearer interpretation of effect variation. Heterogeneity was assessed using the *Q* statistic and *I*². The *Q* test was performed to assess the null hypothesis of homogeneity (ie, that all studies shared a common effect size), with a significance threshold of *α*=.10 to account for its typically low statistical power. *I*² quantified the proportion of observed variance attributable to true heterogeneity rather than random error. However, as the *I*² index does not describe the magnitude of variation in effect sizes, we place primary emphasis on the prediction interval to interpret the distribution of true effects across different populations and settings. Given that estimates of heterogeneity based on fewer than 10 studies may be unreliable, the results should be interpreted with caution. Subgroup analysis and meta-regression were not conducted because the number of available studies was insufficient for reliable moderator testing. Egger regression test and Duval and Tweedie trim and fill procedure were conducted to examine potential dissemination bias. Potential publication bias was assessed within the same random-effects framework in R. To assess the robustness of the findings, a leave-one-out sensitivity analysis was conducted, in which the overall effect was recalculated after omitting each study in turn.

The risk of bias in the included studies was assessed using the revised Cochrane risk-of-bias 2 tool for randomized trials [70]. Three reviewers participated in the assessment process. Each study was evaluated across five domains: (1) bias arising from the randomization process, (2) bias due to deviations from intended interventions, (3) bias due to missing outcome data, (4) bias in measurement of the outcome, and (5) bias in selection of the reported result. Any discrepancies were resolved through joint discussion until a consensus was reached. The overall risk-of-bias judgment for each study followed the standard Cochrane risk-of-bias 2 algorithm, where the highest risk level identified in any individual domain determined the overall rating. The visualization of the risk of bias assessment was produced using the *robvis* tool [71].

The certainty of evidence for the primary outcome (loneliness) was assessed using the GRADE approach [72]. Two reviewers independently performed the assessment, with any discrepancies resolved through discussion until a consensus was reached. We evaluated 5 domains: risk of bias, inconsistency, indirectness, imprecision, and publication bias. The certainty of evidence was graded as high, moderate, low, or very low, reflecting the degree of confidence that the true effect lies close to the estimate. The findings were summarized following the standard GRADE Summary of Findings table format.

## Results

### Study Selection

**Figure 1.**
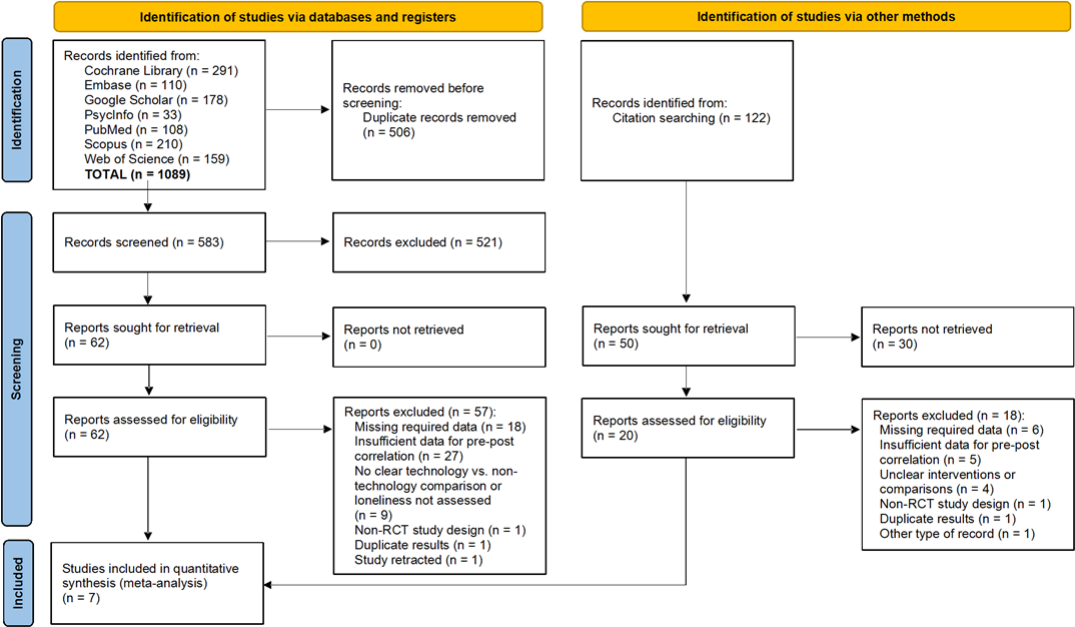
PRISMA (Preferred Reporting Items for Systematic Reviews and Meta-Analyses) flowchart illustrating the study selection process for the systematic review and meta-analysis of technology-based interventions for loneliness (literature search conducted up to August 2024). RCT: randomized controlled trial.

The database search yielded 1089 records. After screening titles and abstracts, full-text reports were assessed for eligibility. Detailed numbers of excluded records at each stage are presented in the PRISMA flow diagram (Figure 1). A total of 57 studies were excluded, primarily due to incomplete or insufficient data. A full list of excluded studies with reasons is provided in Multimedia Appendix 2. Notably, the study by Sun [73], which was initially considered, was excluded due to its retraction by the publisher [74].

Additional studies were sought through citation searching of thematically relevant reviews (n=32). This search yielded 122 records, of which 72 duplicates were removed. Following the assessment of the remaining reports, most were excluded for reasons similar to the database search results, ultimately leaving 2 studies for inclusion (Figure 1). Moreover, to ensure the comprehensiveness of our study selection, we compared our included studies with those identified in the meta-analysis by Jin et al [75], which examined the effectiveness of technology-based interventions for reducing loneliness in older adults. Given the overlap in scope, we reviewed all 6 studies included in their meta-analysis. However, none of them met our strict inclusion criteria. Notably, although the study by Shapira et al [76] was classified as an RCT in the review by Jin et al [75], our assessment revealed that the allocation was based on participants’ willingness and interest rather than randomization. Therefore, it was excluded as a non-RCT. Four other studies [77-80] were excluded due to lacking essential source data or statistical indicators needed for calculating pre-post correlations. Similarly, the study of Slegers et al [81] lacked a clearly defined comparison between a technology-based and a nontechnology-based intervention.

Importantly, the study by Schwindenhammer [82], identified through citation searching, was originally described by the author as having a quasi-experimental design. However, a detailed examination of the methodology revealed that the assignment of nursing homes to intervention or control groups was determined by a random drawing. Consequently, this study qualifies as a cluster-RCT rather than a nonrandomized quasi-experiment. Since the study satisfied the randomization requirement and provided the necessary statistical indicators for calculating pre-post correlations, it was included in the final analysis. Ultimately, 7 studies were included in the quantitative synthesis.

### Study Characteristics

Tables1  2 summarize the characteristics of the 7 included studies, including participant demographics, intervention details, descriptive statistics, and methodological features. The studies included in the meta-analysis were published between 2006 and 2024 and were conducted in the United States (n=3), Switzerland (n=1), Poland (n=1), New Zealand (n=1), and Israel (n=1). The target populations were diverse, ranging from high school students [83] and young adults [84] to adults [85], chronically ill rural women [86], and older adults residing in the community or nursing homes [82,87,88]. Participant ages ranged from 14 to 100 years.

**Table 1.** Characteristics of included studies: participant demographics and settings.

Study	Country	Sample characteristics	Sample N (IG^a^/CG^b^)	Age range (y)	Age, mean (SD)	Gender (% female)
Brog et al [85]	Switzerland	Adults with at least mild depressive symptoms	Randomized: 107; analyzed: 95	18‐81	40.36 (14.59)	81.3
Hill et al [86]	United States	Chronically ill rural women	Randomized: 120; analyzed: 100	35‐65	N/A	100
Iyer et al [83]	United States	High school students	Randomized: 110; analyzed: 108	14‐19	16.05 (1.34)^c^	81.5
Karkosz et al [84]	Poland	Young adults with subclinical depression or anxiety	Randomized: 81; analyzed: 81	18‐35	25.7 (4.59)^c^	71.6
Robinson et al [87]	New Zealand	Older adults in hospital and rest home care	Randomized: 40; analyzed: 34	55‐100	N/A^d^	67.5
Shapira et al [88]	Israel	Older community-dwelling adults	Randomized: 86; analyzed: 82	65‐90	72 (5.63)	80.5
Schwindenhammer [82]	United States	Older adults in nursing homes	Cluster-randomized: 5 nursing homes (83 residents); analyzed: 80	71‐97	86.12 (5.28)	87.5

a IG: intervention group.

b CG: control group.

c Computed from the dataset available, not provided in the study itself.

d Not applicable.

**Table 2.** Characteristics of included studies: intervention details and descriptive outcomes.

Study	Baseline		Postintervention		Measurement instrument	Intervention type	Control type	Intervention duration (wk)	Difference between baseline and follow-up measurement
	N	Mean (SD)	N	Mean (SD)					
Brog et al [85]	IG^a^: 53; CG^b^: 54	IG: 21.26 (4.82); CG: 20.37 (4.25)	IG: 43; CG: 52	IG: 19.88 (4.56); CG: 20.27 (4.04)	UCLA^c^ (9-item)	Internet-based self-help program for psychological distress (CBT)^d^	Wait-list control (care as usual)	3	3 wk
Hill et al [86]	IG: 43; CG: 57	IG: 45.73 (9.99); CG: 43.97 (10.53)	IG: 43; CG: 57	IG: 43.15 (8.69); CG: 43.11 (10.94)	UCLA (20-item)	Online, asynchronous, peer-led support group and health teaching units	No intervention (usual care)	22	3 mo
Iyer et al [83]	IG: 54; CG: 54	IG: 46.54 (10.31); CG: 43.93 (10.97)	IG: 54; CG: 54	IG: 38.07 (8.44); CG: 42.89 (11.49)	UCLA (20-item)	Online, self-care program	Wait-list control	4	4 wk
Karkosz et al [84]	IG: 40; CG: 41	IG: 45.44 (12.15); CG: 42.39 (13.25)	IG: 40; CG: 41	IG: 43.06 (13.38); CG: 41.49 (13.42)	UCLA (20-item)	Web-based and mobile therapy chatbot (CBT)	Active control (self-help book)	2	2 wk
Robinson et al [87]	IG: 17; CG: 17	IG: 36.44 (9.76); CG: 31.71 (9.50)	IG: 17; CG: 17	IG: 32.23 (9.92); CG: 33.93 (8.52)	UCLA (20-item)	Companion robot	Active control (standard activities)	12	12 wk
Shapira et al [88]	IG: 68; CG: 18	IG: 5.43 (2.0); CG: 6.11 (1.9)	IG: 64; CG: 18	IG: 4.80 (1.70); CG: 6.40 (1.70)	UCLA (3-item)	Online guided group sessions via Zoom (CBT)	Wait-list control	3.5	3.5 wk
Schwindenhammer [82]	IG: 40; CG: 40	IG: 17.44 (4.63); CG: 16.45 (4.43)	IG: 40; CG: 40	IG: 14.53 (4.23); CG: 14.13 (3.88)	UCLA (10-item)	Videoconferencing sessions	No intervention (usual care)	14	14 wk

a IG: intervention group.

b CG: control group.

c UCLA: University of California, Los Angeles Loneliness Scale.

d CBT: cognitive behavioral therapy.

The interventions were categorized into 3 distinct types based on their primary mechanism of action and content: (1) digital psychological interventions (n=4), which used technology to deliver structured therapeutic content (eg, cognitive behavioral therapy [CBT], mindfulness) or skills training. This category included a web-based self-help program [85], an online self-care program [83], a therapy chatbot [84], and online guided group CBT sessions [88]. (2) Information and Communication Technology for social connection (n=2), which focused on facilitating communication with others. This included videoconferencing with family members [82] and an online peer-support group [86]. (3) Social robots (n=1), represented by a companion seal robot designed to provide companionship [87].

The duration of interventions varied considerably, ranging from 2 weeks [84] to 22 weeks [86]. All studies assessed loneliness using the University of California, Los Angeles Loneliness Scale, though different versions were used (eg, 3-item, 9-item, 10-item, or 20-item versions). Baseline loneliness scores indicated varying levels of loneliness across samples, from low to moderate to high.

### Results of Syntheses

The meta-analysis demonstrated a small and statistically nonsignificant overall effect of interventions on loneliness (SMD=−0.21, 95% CI −0.59 to 0.17; *P*=.22), indicating that the interventions did not produce a reliable reduction in loneliness compared with control conditions (Table 3). The random-effects model was used to account for between-study heterogeneity, which was moderate (Q=13.68, df=6; *P*=.03; *I*²=57.44%, τ²=0.076; *τ*=0.276), suggesting that a substantial proportion of the variability reflects true differences among studies rather than sampling error.

**Table 3.** Summary of random-effects meta-analysis results, showing standardized mean differences and statistical indicators for loneliness reduction in technology-based interventions vs control groups.

Study	Standardized mean difference	SE	Variance	95% CI	*Z* value	*P* value
Brog et al [85]	−0.019	0.208	0.043	−0.428 to 0.389	−0.093	.93
Hill et al [86]	−0.049	0.209	0.043	−0.457 to 0.360	−0.234	.82
Iyer et al [83]	0	0.212	0.045	−0.416 to 0.416	0.000	>.99
Karkosz et al [84]	−0.003	0.227	0.051	−0.448 to 0.441	−0.015	.99
Robinson et al [87]	−1.047	0.366	0.134	−1.764 to −0.330	−2.863	.004
Schwindenhammer [82]	0	0.231	0.053	−0.453 to 0.453	0.000	>.99
Shapira et al [88]	−0.824	0.282	0.080	−1.378 to −0.271	−2.919	.004

**Figure 2.**
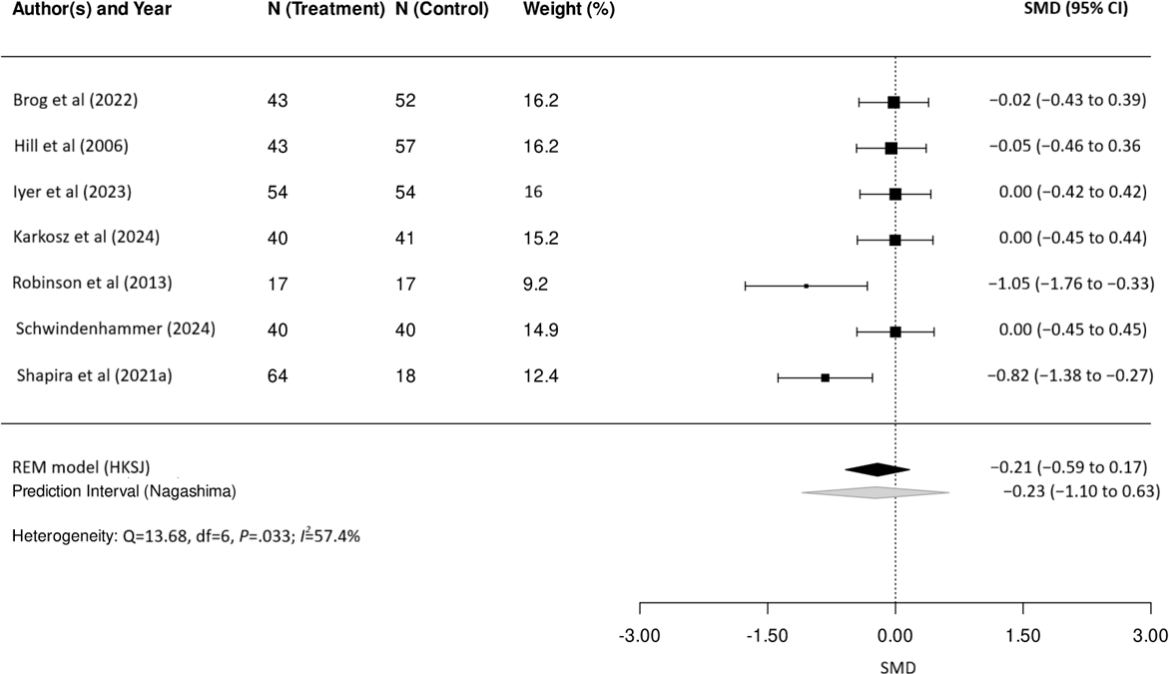
Forest plot showing study-level standardized mean differences and 95% CIs for the effect of technology-based interventions on loneliness, synthesized using a Hartung-Knapp-Sidik-Jonkman adjusted random-effects model [82-88]. HKSJ: Hartung-Knapp-Sidik-Jonkman method; *I*²: *I*-squared heterogeneity statistic; Q: Cochran Q statistic; REML: restricted maximum-likelihood; SMD: standardized mean difference.

The estimated prediction interval ranges from –1.14 to 0.63, indicating that true effects in comparable future studies may vary from modest reductions to modest increases in loneliness (Figure 2). Given that the analysis included only 7 studies, heterogeneity estimates and the prediction interval should be interpreted with caution. Further research is needed to better identify conditions under which loneliness interventions are effective.

### Risk of Bias

**Figure 3.**
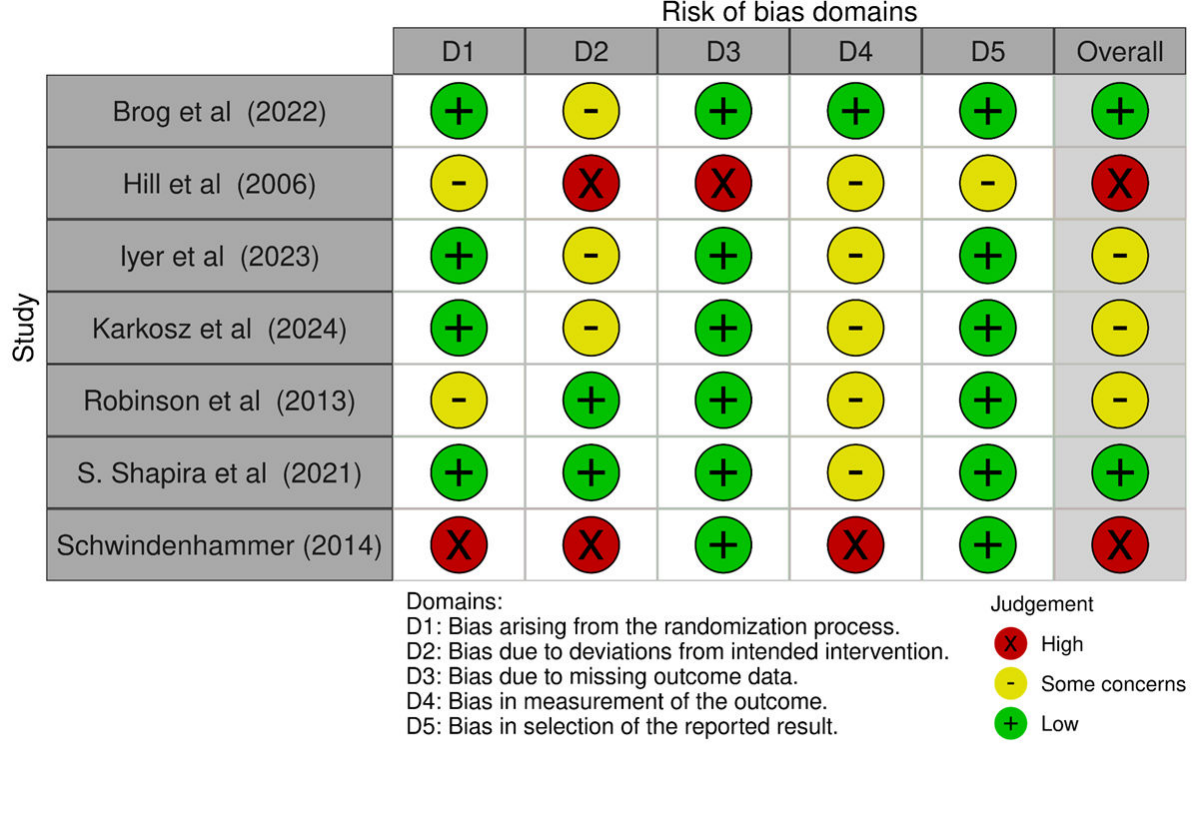
Risk of bias summary: review authors’ judgments about each risk of bias item for each included study [82-88].

The summary of the risk of bias assessment is presented in Figure 3. Among the 7 included studies, 2 were judged to be at low risk of bias [85,88], 3 studies raised some concerns [83,84,87], and 2 studies were assessed as being at high risk of bias [82,86]. The high risk was primarily driven by domain 3 (missing outcome data) due to substantial differential attrition between the intervention and control groups [86] and domain 4 (measurement of the outcome) due to the lack of blinding of the outcome assessor during data collection [82]. Most studies raised “some concerns” in domain 2 (deviations from intended interventions) and domain 4. This reflects the inherent nature of psychosocial and technological interventions, where blinding of participants is often not feasible, and the reliance on self-reported measures may introduce bias if the participants are aware of their group assignment.

### Reporting Biases

Visual inspection of the funnel plot and statistical testing were used to evaluate reporting bias. Egger regression test [89] showed statistically significant funnel plot asymmetry (*t*_5_=–5.47, *P*=.003), indicating the presence of small-study effects (see Funnel Plot in Multimedia Appendix 3). However, Duval and Tweedie trim and fill method [90] did not identify any missing studies (0 missing studies), and no adjusted pooled effect was required. Accordingly, although funnel plot asymmetry was detected, the absence of imputed studies suggests that the asymmetry is likely driven by study heterogeneity (as indicated by the *I*^2^ and wide prediction interval) rather than publication bias alone.

### Sensitivity Analysis

A sensitivity analysis was conducted using a leave-one-out procedure in R, in which the pooled effect was recalculated after omitting each study in turn. The results showed that the pooled SMD remained small and nonsignificant across all iterations, and the direction of the effect did not change, indicating that no single study unduly influenced the overall findings.

### Certainty of Evidence

The overall certainty of the evidence regarding the effect of technology-based interventions on loneliness was graded as very low. We downgraded the evidence by 1 level due to the risk of bias, as only 2 of the 7 included studies were rated as having a low risk of bias. We further downgraded the evidence due to inconsistencies caused by moderate heterogeneity and a wide prediction interval. Finally, we downgraded the rating due to imprecision, as the 95% CI for the pooled effect included both a noticeable benefit and no effect (crossing the 0 line). No publication bias was detected, but given the small number of studies, it could not be completely ruled out. A detailed summary of the findings and certainty assessment is provided in Multimedia Appendix 4.

## Discussion

### Principal Findings

The primary objective of this meta-analysis was to evaluate the effectiveness of technology-based interventions in reducing loneliness. The results showed a small and statistically nonsignificant overall effect, indicating that the available evidence does not support a reliable reduction in loneliness attributable to technology-based interventions. Importantly, these findings must be interpreted with caution, as the certainty of the evidence was graded as very low. This low confidence stems primarily from limitations in the risk of bias domain and the observed inconsistency across studies. However, the moderate heterogeneity observed in the studies suggests that the effectiveness of these interventions varies across studies. The prediction interval indicated that true effects in comparable future studies could range from substantial reductions to moderate increases in loneliness, underscoring the influence of study-specific and contextual factors.

Because the number of available studies (k=7) did not permit reliable subgroup or moderator analyses, the present meta-analysis could not empirically evaluate how different types of technology-based interventions, such as digital psychological interventions or information and communication technology for social connection, vary in their effectiveness. However, previous research provides important insights into potential sources of variability. For example, some structured approaches, such as telephone-delivered behavioral activation or mindfulness interventions, have demonstrated beneficial effects compared with less structured social-contact interventions [89], where a telephone-based behavioral activation or mindfulness intervention is effective compared with a control group that receives only a befriending intervention [89]. Moreover, classifying interventions by mechanism of action rather than just delivery mode offers insights for future research. For example, digital psychological interventions aiming to restructure maladaptive social cognition (eg, via CBT) have previously shown strong effects in face-to-face settings [28]. In our review, studies in this category [84,85,88] showed mixed results, potentially due to variations in guidance intensity. Research suggests that loneliness is a deeply personal experience shaped by social, cultural, and psychological factors [8,9]. As a result, intervention effectiveness is likely to depend on participant characteristics such as baseline loneliness, age, social resources, and psychological needs, although these factors could not be tested in the present analysis. Prior work suggests that digital mental health interventions are more effective when tailored to the needs and preferences of individuals [91], underscoring the importance of personalization rather than a uniform intervention model.

Despite the widespread interest in technology-based interventions, it is important to acknowledge the moderate proportion of heterogeneity in effects across studies. We observed an *I*² of 57%, indicating that more than half of the observed variance in effect sizes is due to true differences between studies rather than sampling error. This finding is consistent with prior observations that loneliness intervention outcomes are highly context-dependent [92-94]. However, the *I*² statistic does not reveal by how much the true effect varies across different populations. The prediction interval, in contrast, provides a direct estimate of this range. Previous reviews of loneliness interventions have also frequently reported substantial heterogeneity [28,95,96]. This variability may be due to differences in study design, with pre-post studies and nonrandomized comparative studies showing larger average effect sizes compared to randomized controlled studies [28]. The sources of heterogeneity could not be examined empirically in the present meta-analysis due to the small number of included studies; however, prior research suggests that study design, participant age, and digital literacy may influence intervention outcomes [97]. For example, younger participants often benefit more from digital programs that incorporate interactive or socially engaging elements, whereas older adults may experience additional barriers such as lower digital literacy or chronic social isolation that limit intervention impact [91,98-100]. Systematic reviews focusing specifically on older adults have reported minimal or no overall effects of digital interventions [75,101], underscoring the importance of population characteristics. Previous literature also indicates that intervention type, delivery format, and follow-up duration may influence loneliness outcomes [102-104], suggesting that program content and user engagement processes play important roles. Additionally, effects may vary by timing of assessment, with short-term improvements sometimes diminishing over longer follow-up periods [101,104]. Lastly, differences in loneliness measurement instruments and study quality cannot be overlooked. Most studies in the broader literature use the University of California, Los Angeles Loneliness Scale, but other instruments, including single-item measures, are also used [105,106]. Meta-analytic evidence indicates that effect sizes can differ by instrument, partly due to scale sensitivity and range [107-110]. The use of a random-effects model helps account for some of this variability; however, the prediction interval we calculated in the present study was wide. This highlights a critical distinction: while the confidence interval suggests a nonsignificant average effect, the prediction interval implies that in specific future settings, a technology-based intervention might show only a negligible effect on loneliness or, conversely, a somewhat larger effect than the pooled estimate suggests.

A significant challenge encountered in this meta-analysis was the lack of essential data required for the computation of the effect size. Many studies failed to report sufficient statistical details, which considerably reduced the number of studies that could ultimately be included in the meta-analysis. This limitation was particularly striking given the large number of studies initially identified as suitable during the preliminary screening phase. Furthermore, some studies provided links to external repositories, where relevant data were supposed to be available. However, upon verification, these datasets were either missing or incomplete. This issue emphasizes the critical importance of comprehensive and transparent reporting in research. Consistent and standardized reporting of results, including the provision of complete datasets, would greatly enhance the feasibility and reliability of future meta-analyses and systematic reviews [63,111]. More rigorous data availability would allow for more robust conclusions with greater explanatory value, and so improve the overall quality and impact of evidence synthesis in the field.

Our meta-analysis found a small and statistically nonsignificant overall effect of technology-based interventions on loneliness, which should be interpreted in the context of prior research on loneliness interventions. Earlier meta-analyses in the field have generally reported small-to-moderate effects. For example, the landmark review by Masi et al [28], which examined various approaches such as social skills training, enhanced social support, and opportunities for social contact, found overall small reductions in loneliness, with the strongest effects observed for interventions addressing maladaptive social cognition [28,92]. In a more recent comprehensive review, Lasgaard et al [24] synthesized 128 studies and likewise reported a moderate loneliness reduction for randomized trials (SMD on the order of ~ −0.5). These earlier findings provide important context for interpreting our more modest pooled effect, and reinforce the conclusion that loneliness interventions in general tend to yield small-to-moderate benefits. Indeed, when focusing on digital interventions for older adults, recent meta-analyses have often found no significant effect: both Jin et al [75] and Shah et al [101] concluded that technology-based programs did not measurably reduce loneliness in older populations (pooled SMDs around 0 to −0.1; *P*>.5). An earlier analysis by Choi et al [112] had suggested that computer and internet training programs can be effective for older adults, but the evidence base at that time was limited to a few small studies. Given that the present meta-analysis spans a broader age range, its findings contribute to a more nuanced picture of how digital interventions may operate across different demographic groups. The literature has yielded mixed results depending on population characteristics and intervention modality [24,28,103], and while such comparisons could not be undertaken in the present study, prior findings highlight the value of considering demographic and contextual factors when evaluating intervention outcomes. For instance, Eccles and Qualter [23] examined interventions among children and adolescents and found that while youth loneliness can be alleviated, effects varied greatly between studies, with substantial unexplained heterogeneity. Taken together, the broader evidence base suggests that intervention effectiveness may depend on factors such as target population and intervention format, though these influences could not be directly assessed in the present meta-analysis.

### Strengths and Limitations

This meta-analysis adheres to PRISMA guidelines, ensuring methodological transparency and rigor. A comprehensive search strategy across multiple databases, supplemented by citation tracking, minimizes the risk of missing relevant studies. By incorporating prediction intervals alongside standard heterogeneity assessments, the analysis provides deeper insights into the variability of intervention effects. Furthermore, it contributes to the growing evidence base on technology-based interventions for loneliness, which is particularly relevant in the context of increasing workforce shortages in health care and social services.

However, the study has several limitations. First, the number of eligible studies that provided sufficient statistical information was small, which restricted the scope of the meta-analysis and limited the ability to conduct subgroup or moderator analyses. This constrains the conclusions that can be drawn regarding factors that may influence intervention effectiveness. Furthermore, several limitations of the review process should be acknowledged. Although we did not use formal language restrictions, we used English keywords for the search. This may have limited the identification of studies published in other languages. Additionally, our search strategy relied on major databases and citation tracking. We did not conduct a separate systematic search for gray literature, so some unpublished trials might have been missed. We also decided not to contact study authors for missing data because of the practical reasons explained in the *Methods* section. This resulted in the exclusion of studies that could have been eligible if more information was available.

### Implications

Technology is not a panacea for loneliness, yet it may serve as a useful tool when applied meaningfully and with consideration for the individual needs of the user. Communication technologies, such as video calls and messaging platforms, have been proposed as complements to interpersonal relationships and may help reduce feelings of loneliness in some contexts, particularly among older adults [113]. Similarly, social robots have emerged as nonpharmacological interventions capable of reducing both loneliness and depression [114]. However, a critical factor remains the manner in which these technologies are used. Active engagement in digital interactions has been shown to yield positive effects, whereas passive consumption of digital content may, conversely, exacerbate feelings of isolation [115]. Therefore, it is essential to consider users’ age, technological literacy, and personal preferences when implementing technological solutions, to maximize their potential benefits and to avoid unintended increases in loneliness or social isolation.

### Conclusions

This meta-analysis provides a rigorous synthesis of RCTs evaluating technology-based interventions for loneliness, including participants from adolescence to older adulthood. This study differs from existing reviews by focusing exclusively on randomized trials and the inclusion of a broader age demographic, whereas previous landmark studies often combined different study designs [28] or focused specifically on the older adult population [75]. Furthermore, the use of an RCT-only design, Hartung-Knapp adjustment, and prediction intervals offers a rigorous summary of the current evidence and highlights substantial variability in intervention outcomes. The small and nonsignificant pooled effect, together with moderate heterogeneity and a wide prediction interval, shows that results differ across studies and settings. These findings clarify current limitations in the evidence base and point to the need for better-designed and more transparently reported trials. In practice, technology-based approaches should be considered supportive tools that require careful tailoring to user characteristics and implementation contexts rather than universal solutions to loneliness.

## Supplementary material

10.2196/80059 Multimedia Appendix 1 Search syntaxes for database search.

10.2196/80059 Multimedia Appendix 2 List of excluded studies.

10.2196/80059 Multimedia Appendix 3 Funnel plot.

10.2196/80059 Multimedia Appendix 4 GRADE (Grading of Recommendations Assessment, Development, and Evaluation) summary of findings: technology-based interventions compared to control conditions for reducing loneliness.

10.2196/80059 Checklist 1 PRISMA 2020 checklist, PRISMA 2020 for Abstracts checklist, and PRISMA-S checklist.

## Abbreviations

CBT: cognitive behavioral therapy
GRADE: Grading of Recommendations Assessment, Development, and Evaluation
PRISMA: Preferred Reporting Items for Systematic Reviews and Meta-Analyses
PRISMA-S: Preferred Reporting Items for Systematic Reviews and Meta-Analyses-Search extension
PROSPERO: International Prospective Register of Systematic Reviews
RCT: randomized controlled trial
SMD: standardized mean difference
