# Supplementary material for: Efficacy of Technology-Based Interventions on the Reduction of Loneliness: Systematic Review and Meta-Analysis
Source: J Med Internet Res. 2026 May 8;28:e80059. doi: 10.2196/80059 (PMC13158556; doi:10.2196/80059)
Supplement: Multimedia Appendix 1 [file jmir-v28-e80059-s001.docx]

# Multimedia Appendix 1

## Search syntaxes for database search

### Web of Science

TS=((loneliness OR "perceived social isolation" OR "perceived emotional isolation") AND (intervention OR treatment OR therapy) AND (technolog* OR software OR computer OR web OR smart* OR mobile OR cellphone OR internet OR "artificial intelligence" OR app OR apps OR appl* OR video*) AND ("randomized controlled trial" OR "randomised controlled trial" OR RCT))

### PsycInfo

( TI ( loneliness OR "perceived social isolation" OR "perceived emotional isolation" ) AND TI ( intervention OR treatment OR therapy ) AND TI ( technolog* OR software OR computer OR web OR smart* OR mobile OR cellphone OR internet OR "artificial intelligence" OR app OR apps OR appl* OR video* ) AND TI ( "randomized controlled trial" OR "randomised controlled trial" OR rct ) ) OR ( AB ( loneliness OR "perceived social isolation" OR "perceived emotional isolation" ) AND AB ( intervention OR treatment OR therapy ) AND AB ( technolog* OR software OR computer OR web OR smart* OR mobile OR cellphone OR internet OR "artificial intelligence" OR app OR apps OR appl* OR video* ) AND AB ( "randomized controlled trial" OR "randomised controlled trial" OR rct ) ) OR ( KW ( loneliness OR "perceived social isolation" OR "perceived emotional isolation" ) AND KW ( intervention OR treatment OR therapy ) AND KW ( technolog* OR software OR computer OR web OR smart* OR mobile OR cellphone OR internet OR "artificial intelligence" OR app OR apps OR appl* OR video* ) AND KW ( "randomized controlled trial" OR "randomised controlled trial" OR rct ) )

### PubMed

(loneliness[Title/Abstract] OR "perceived social isolation"[Title/Abstract] OR "perceived emotional isolation"[Title/Abstract]) AND (intervention[Title/Abstract] OR treatment[Title/Abstract] OR therapy[Title/Abstract]) AND ("randomized controlled trial"[Title/Abstract] OR "randomised controlled trial"[Title/Abstract] OR RCT[Title/Abstract]) AND (technolog*[Title/Abstract] OR software[Title/Abstract] OR computer[Title/Abstract] OR web[Title/Abstract] OR smart*[Title/Abstract] OR mobile[Title/Abstract] OR cellphone[Title/Abstract] OR internet[Title/Abstract] OR "artificial intelligence"[Title/Abstract] OR app[Title/Abstract] OR apps[Title/Abstract] OR appl*[Title/Abstract] OR video*[Title/Abstract])

Initially, the search syntax included only the term "app*". However, this was subsequently revised to "app OR apps OR appl*" for two reasons: (1) PubMed does not support searches for terms with only three characters followed by an asterisk (e.g., "app*"), and (2) the string "app*" captures unrelated terms such as "approach" or "appropriate," which are not relevant to the scope of this review.

### Scopus

( TITLE-ABS-KEY ( loneliness OR {perceived social isolation} OR {perceived emotional isolation} ) AND TITLE-ABS-KEY ( intervention OR treatment OR therapy ) AND TITLE-ABS-KEY ( {randomized controlled trial} OR {randomised controlled trial} OR rct ) AND TITLE-ABS-KEY ( technolog* OR software OR computer OR web OR smart* OR mobile OR cellphone OR internet OR {artificial intelligence} OR app OR apps OR appl* OR video* ) )

### Google Scholar

Intext:((loneliness OR "perceived social isolation" OR "perceived emotional isolation") AND (intervention OR treatment OR therapy) AND ("randomized Controlled Trial" OR "randomised Controlled Trial" OR RCT) AND (technolog* OR software OR computer OR web OR smart* OR mobile OR cellphone OR internet OR "artificial intelligence" OR app OR apps OR appl* OR video*))

Due to Google Scholar’s limitations in search syntax, searches were conducted using the *intext:* operator, which scans the full text for specified terms. Because of a character limit (256 characters), the last bracketed group of terms (technology-related keywords) was split into 13 separate searches. Duplicates were subsequently removed using the Zotero software.

### Embase

(loneliness:ti,ab,kw OR 'perceived social isolation':ti,ab,kw OR 'perceived emotional isolation':ti,ab,kw) AND (intervention:ti,ab,kw OR treatment:ti,ab,kw OR therapy:ti,ab,kw) AND (technolog*:ti,ab,kw OR software:ti,ab,kw OR computer:ti,ab,kw OR web:ti,ab,kw OR smart*:ti,ab,kw OR mobile:ti,ab,kw OR cellphone:ti,ab,kw OR internet:ti,ab,kw OR 'artificial intelligence':ti,ab,kw OR app:ti,ab,kw OR apps:ti,ab,kw OR appl*:ti,ab,kw OR video*:ti,ab,kw) AND ('randomized controlled trial':ti,ab,kw OR 'randomised controlled trial':ti,ab,kw OR rct:ti,ab,kw)

### Cochrane Library

(loneliness OR "perceived social isolation" OR "perceived emotional isolation") AND (intervention OR treatment OR therapy) AND (technolog* OR software OR computer OR web OR smart* OR mobile OR cellphone OR internet OR "artificial intelligence" OR app OR apps OR appl* OR video*) AND ("randomized controlled trial" OR "randomised controlled trial" OR RCT) in Title Abstract Keyword - (Word variations have been searched)

## Review articles for citation searching

Andersson, G. (2024). Innovating CBT and Answering New Questions: The Role of Internet-Delivered CBT. *International Journal of Cognitive Therapy*, *17*(2), 179–190. <https://doi.org/10.1007/s41811-023-00199-5>

Astasio-Picado, Á., Cobos-Moreno, P., Gómez-Martín, B., Verdú-Garcés, L., & Zabala-Baños, M. del C. (2022). Efficacy of Interventions Based on the Use of Information and Communication Technologies for the Promotion of Active Aging. *International Journal of Environmental Research and Public Health*, *19*(3), 1534. <https://doi.org/10.3390/ijerph19031534>

Balcombe, L., & De Leo, D. (2023). Evaluation of the Use of Digital Mental Health Platforms and Interventions: Scoping Review. *International Journal of Environmental Research and Public Health*, *20*(1), 362. <https://doi.org/10.3390/ijerph20010362>

Bixter, M. T., Blocker, K. A., & Rogers, W. A. (2018). 8—Enhancing social engagement of older adults through technology. *Aging, Technology and Health*, 179–214. <https://doi.org/10.1016/B978-0-12-811272-4.00008-7>

Borghouts, J., Eikey, E., Mark, G., Leon, C. D., Schueller, S. M., Schneider, M., Stadnick, N., Zheng, K., Mukamel, D., & Sorkin, D. H. (2021). Barriers to and Facilitators of User Engagement With Digital Mental Health Interventions: Systematic Review. *Journal of Medical Internet Research*, *23*(3), e24387. <https://doi.org/10.2196/24387>

Cuijpers, P., van Straten, A., & Andersson, G. (2008). Internet-administered cognitive behavior therapy for health problems: A systematic review. *Journal of Behavioral Medicine*, *31*(2), 169–177. <https://doi.org/10.1007/s10865-007-9144-1>

Dickens, A. P., Richards, S. H., Greaves, C. J., & Campbell, J. L. (2011). Interventions targeting social isolation in older people: A systematic review. *BMC Public Health*, *11*(1), 647. <https://doi.org/10.1186/1471-2458-11-647>

Dworschak, C., Heim, E., & Maercker, A. (2022). Efficacy of internet-based interventions for common mental disorder symptoms and psychosocial problems in older adults: A systematic review and meta-analysis. *Internet Interventions*, *27*, 100498. <https://doi.org/10.1016/j.invent.2022.100498>

Ellard, O. B., Dennison, C., & Tuomainen, H. (2023). Review: Interventions addressing loneliness amongst university students: a systematic review. *Child and Adolescent Mental Health*, *28*(4), 512–523. <https://doi.org/10.1111/camh.12614>

Gorenko, J. A., Moran, C., Flynn, M., Dobson, K., & Konnert, C. (2021). Social Isolation and Psychological Distress Among Older Adults Related to COVID-19: A Narrative Review of Remotely-Delivered Interventions and Recommendations. *Journal of Applied Gerontology*, *40*(1), 3–13. <https://doi.org/10.1177/0733464820958550>

Hagan, R., Manktelow, R., Taylor, B. J., & Mallett, J. (2014). Reducing loneliness amongst older people: A systematic search and narrative review. *Aging & Mental Health*, *18*(6), 683–693. <https://doi.org/10.1080/13607863.2013.875122>

Hao, X., Qin, Y., Lv, M., Zhao, X., Wu, S., & Li, K. (2023). Effectiveness of telehealth interventions on psychological outcomes and quality of life in community adults during the COVID-19 pandemic: A systematic review and meta-analysis. *International Journal of Mental Health Nursing*, *32*(4), 979–1007. <https://doi.org/10.1111/inm.13126>

Heins, P., Boots, L. M. M., Koh, W. Q., Neven, A., Verhey, F. R. J., & de Vugt, M. E. (2021). The Effects of Technological Interventions on Social Participation of Community-Dwelling Older Adults with and without Dementia: A Systematic Review. *Journal of Clinical Medicine*, *10*(11), 2308. <https://doi.org/10.3390/jcm10112308>

Hickin, N., Käll, A., Shafran, R., Sutcliffe, S., Manzotti, G., & Langan, D. (2021). The effectiveness of psychological interventions for loneliness: A systematic review and meta-analysis. *Clinical Psychology Review*, *88*, 102066. <https://doi.org/10.1016/j.cpr.2021.102066>

Hoang, P., King, J. A., Moore, S., Moore, K., Reich, K., Sidhu, H., Tan, C. V., Whaley, C., & McMillan, J. (2022). Interventions Associated With Reduced Loneliness and Social Isolation in Older Adults: A Systematic Review and Meta-analysis. *JAMA Network Open*, *5*(10), e2236676. <https://doi.org/10.1001/jamanetworkopen.2022.36676>

Chang, H., Do, Y., & Ahn, J. (2023). Digital Storytelling as an Intervention for Older Adults: A Scoping Review. *International Journal of Environmental Research and Public Health*, *20*(2), 1344. <https://doi.org/10.3390/ijerph20021344>

Ibarra, F., Baez, M., Cernuzzi, L., & Casati, F. (2020). A Systematic Review on Technology-Supported Interventions to Improve Old-Age Social Wellbeing: Loneliness, Social Isolation, and Connectedness. *Journal of Healthcare Engineering*, *2020*(1), 2036842. <https://doi.org/10.1155/2020/2036842>

Jin, W., Liu, Y., Yuan, S., Bai, R., Li, X., & Bai, Z. (2021). The Effectiveness of Technology-Based Interventions for Reducing Loneliness in Older Adults: A Systematic Review and Meta-Analysis of Randomized Controlled Trials. *Frontiers in Psychology*, *12*. <https://doi.org/10.3389/fpsyg.2021.711030>

Morris, M. E., Adair, B., Ozanne, E., Kurowski, W., Miller, K. J., Pearce, A. J., Santamaria, N., Long, M., Ventura, C., & Said, C. M. (2014). Smart technologies to enhance social connectedness in older people who live at home. *Australasian Journal on Ageing*, *33*(3), 142–152. <https://doi.org/10.1111/ajag.12154>

Noone, C., McSharry, J., Smalle, M., Burns, A., Dwan, K., Devane, D., & Morrissey, E. C. (2020). *Video calls for reducing social isolation and loneliness in older people: A rapid review*. *5*. <https://doi.org/10.1002/14651858.CD013632>

O’Rourke, H. M., Collins, L., & Sidani, S. (2018). Interventions to address social connectedness and loneliness for older adults: A scoping review. *BMC Geriatrics*, *18*(1), 214. <https://doi.org/10.1186/s12877-018-0897-x>

Phang, J. K., Kwan, Y. H., Yoon, S., Goh, H., Yee, W. Q., Tan, C. S., & Low, L. L. (2023). Digital Intergenerational Program to Reduce Loneliness and Social Isolation Among Older Adults: Realist Review. *JMIR Aging*, *6*(1), e39848. <https://doi.org/10.2196/39848>

Pu, L., Moyle, W., Jones, C., & Todorovic, M. (2019). The Effectiveness of Social Robots for Older Adults: A Systematic Review and Meta-Analysis of Randomized Controlled Studies. *The Gerontologist*, *59*(1), e37–e51. <https://doi.org/10.1093/geront/gny046>

Shah, S. G. S., Nogueras, D., Woerden, H. C. van, & Kiparoglou, V. (2021). Evaluation of the Effectiveness of Digital Technology Interventions to Reduce Loneliness in Older Adults: Systematic Review and Meta-analysis. *Journal of Medical Internet Research*, *23*(6), e24712. <https://doi.org/10.2196/24712>

Shekelle, P. G., Miake-Lye, I. M., Begashaw, M. M., Booth, M. S., Myers, B., Lowery, N., & Shrank, W. H. (2024). Interventions to Reduce Loneliness in Community-Living Older Adults: A Systematic Review and Meta-analysis. *Journal of General Internal Medicine*, *39*(6), 1015–1028. <https://doi.org/10.1007/s11606-023-08517-5>

Suragarn, U., Hain, D., & Pfaff, G. (2021). Approaches to enhance social connection in older adults: An integrative review of literature. *Aging and Health Research*, *1*(3), 100029. <https://doi.org/10.1016/j.ahr.2021.100029>

Toh, G., Pearce, E., Vines, J., Ikhtabi, S., Birken, M., Pitman, A., & Johnson, S. (2022). Digital interventions for subjective and objective social isolation among individuals with mental health conditions: A scoping review. *BMC Psychiatry*, *22*(1), 331. <https://doi.org/10.1186/s12888-022-03889-0>

Tong, F., Yu, C., Wang, L., Chi, I., & Fu, F. (2021). Systematic Review of Efficacy of Interventions for Social Isolation of Older Adults. *Frontiers in Psychology*, *12*. <https://doi.org/10.3389/fpsyg.2021.554145>

Veronese, N., Galvano, D., D’Antiga, F., Vecchiato, C., Furegon, E., Allocco, R., Smith, L., Gelmini, G., Gareri, P., Solmi, M., Yang, L., Trabucchi, M., De Leo, D., & Demurtas, J. (2021). Interventions for reducing loneliness: An umbrella review of intervention studies. *Health & Social Care in the Community*, *29*(5), e89–e96. <https://doi.org/10.1111/hsc.13248>

Welch, V., Ghogomu, E. T., Barbeau, V. I., Dowling, S., Doyle, R., Beveridge, E., Boulton, E., Desai, P., Huang, J., Elmestekawy, N., Hussain, T., Wadhwani, A., Boutin, S., Haitas, N., Kneale, D., Salzwedel, D. M., Simard, R., Hébert, P., & Mikton, C. (2023). Digital interventions to reduce social isolation and loneliness in older adults: An evidence and gap map. *Campbell Systematic Reviews*, *19*(4), e1369. <https://doi.org/10.1002/cl2.1369>

Williams, C. Y. K., Townson, A. T., Kapur, M., Ferreira, A. F., Nunn, R., Galante, J., Phillips, V., Gentry, S., & Usher-Smith, J. A. (2021). Interventions to reduce social isolation and loneliness during COVID-19 physical distancing measures: A rapid systematic review. *PLOS ONE*, *16*(2), e0247139. <https://doi.org/10.1371/journal.pone.0247139>

Ye, Z., Li, W., & Zhu, R. (2022). Online psychosocial interventions for improving mental health in people during the COVID-19 pandemic: A systematic review and meta-analysis. *Journal of Affective Disorders*, *316*, 120–131. <https://doi.org/10.1016/j.jad.2022.08.023>
