# Supplementary material for: Efficacy of Technology-Based Interventions on the Reduction of Loneliness: Systematic Review and Meta-Analysis
Source: J Med Internet Res. 2026 May 8;28:e80059. doi: 10.2196/80059 (PMC13158556; doi:10.2196/80059)
Supplement: Multimedia Appendix 2 [file jmir-v28-e80059-s002.docx]

**Supplementary Material 3**

**A list of excluded studies**

| **Year** | **Authors** | **Title** | **Reasons for exclusion** | **Detailed reasons for exclusion** | **Notes on exclusion** |
| --- | --- | --- | --- | --- | --- |
| 2023 | Ae-Ri, Jung; Kowoon, Lee; Eun-A, Park | Development and evaluation of the information and communication technology-based Loneliness Alleviation Program for community-dwelling older adults: A pilot study and randomized controlled trial | insufficient data for pre-post correlation | no publically available data |  |
| 2023 | Althouse, Andrew D.; Abebe, Kaleab Z.; Paasche-Orlow, Michael K.; Lalama, Christina M.; Ferry, Danielle; Lancet, Michelle; Swabe, Gretchen; Bickmore, Timothy; Magnani, Jared W. | Design, rationale, and baseline characteristics of a randomized controlled trial evaluating a mobile relational agent to enhance atrial fibrillation self-care | unrelated topic | no loneliness | loneliness is not an outcome |
| 2023 | Aomori, Maki; Matsumoto, Chiharu; Takebayashi, Sanae; Matsuyama, Nao; Uto, Yukiko; Tanaka, Miho; Samukawa, Sei; Kato, Hideaki; Nakajima, Hideaki; Maeda, Hitomi | Effects of a smartphone app-based diet and physical activity program for men living with HIV who have dyslipidemia: A pilot randomized controlled trial | unrelated topic | no technology | technology only as a supplement to personal consultations |
| 2023 | Arakawa, Yuki; Haseda, Maho; Inoue, Kosuke; Nishioka, Daisuke; Kino, Shiho; Nishi, Daisuke; Hashimoto, Hideki; Kondo, Naoki | Effectiveness of mHealth consultation services for preventing postpartum depressive symptoms: a randomized clinical trial | unrelated topic | no technology | no comparison technology vs no technology |
| 2022 | Beauchet, Olivier; Matskiv, Jacqueline; Galery, Kevin; Goossens, Linda; Lafontaine, Constance; Sawchuk, Kim | Benefits of a 3-month cycle of weekly virtual museum tours in community dwelling older adults: Results of a randomized controlled trial | insufficient data for pre-post correlation | no publically available data |  |
| 2021 | Boekhout, Janet M.; Volders, Esmee; Bolman, Catherine A. W.; de Groot, Renate H. M.; Lechner, Lilian | Long-Term Effects on Loneliness of a Computer-Tailored Intervention for Older Adults With Chronic Diseases: A Randomized Controlled Trial | lack of required data |  | results in B and SE |
| 2024 | Boucher, Eliane M.; Ward, Haley; Miles, Cynthia J.; Henry, Robert D.; Stoeckl, Sarah Elizabeth | Effects of a Digital Mental Health Intervention on Perceived Stress and Rumination in Adolescents Aged 13 to 17 Years: Randomized Controlled Trial | insufficient data for pre-post correlation | no publically available data |  |
| 2019 | Brodbeck, Jeannette; Berger, Thomas; Biesold, Nicola; Rockstroh, Franziska; Znoj, Hans Joerg | Evaluation of a guided internet-based self-help intervention for older adults after spousal bereavement or separation/divorce: A randomised controlled trial | insufficient data for pre-post correlation | no publically available data |  |
| 2020 | Bruehlman-Senecal, Emma; Hook, Cayce J.; Pfeifer, Jennifer H.; FitzGerald, Caroline; Davis, Brittany; Delucchi, Kevin L.; Haritatos, Jana; Ramo, Danielle E. | Smartphone App to Address Loneliness Among College Students: Pilot Randomized Controlled Trial | insufficient data for pre-post correlation | no publically available data |  |
| 2015 | Cleary, Elizabeth H.; Stanton, Annette L. | Mediators of an Internet-Based Psychosocial Intervention for Women With Breast Cancer | lack of required data |  | results in B and SE |
| 2022 | Demirağ, H.; Hintistan, S. | Investigation of physiological and psychological effects of robotic cat and betta fish therapies in hemodialysis patients: A randomized controlled study | lack of required data |  | data in medians and 25th-75th percentile |
| 2009 | Dennis, C-L; Hodnett, E.; Kenton, L.; Weston, J.; Zupancic, J.; Stewart, D. E.; Kiss, A. | Effect of peer support on prevention of postnatal depression among high risk women: multisite randomised controlled trial | lack of required data |  | not detailed loneliness results |
| 2015 | Dodge, Hiroko H.; Zhu, Jian; Mattek, Nora; Bowman, Molly; Ybarra, Oscar; Wild, Katherine; Loewenstein, David A.; Kaye, Jeffrey A. | Web-enabled conversational interactions as a method to improve cognitive functions: Results of a 6-week randomized controlled trial | lack of required data |  | data in M and SD are only in the baseline, then in the form of linear regression results |
| 2024 | Dworschak, Christine; Heim, Eva; Tröster, Alicia; Grunder, Chantal; Maercker, Andreas | Feasibility, acceptability, and preliminary efficacy of an internet-based CBT intervention for loneliness in older adults: A pilot RCT | insufficient data for pre-post correlation | no publically available data |  |
| 2021 | Fields, J.; Cemballi, A.G.; Michalec, C.; Uchida, D.; Griffiths, K.; Cardes, H.; Cuellar, J.; Chodos, A.H.; Lyles, C.R. | In-Home Technology Training Among Socially Isolated Older Adults: Findings From the Tech Allies Program | lack of required data |  | data in regression model format - adjusted odds ratio and CI |
| 2019 | Gustafson, D.H.; Cody, O.J.; Chih, M.-Y.; Johnston, D.C.; Asthana, S. | Pilot Test of a Computer-Based System to Help Family Caregivers of Dementia Patients | lack of required data |  | post-intervention results are in the form of Δ M and SD |
| 2019 | Haslam, Catherine; Cruwys, Tegan; Chang, Melissa X-L; Bentley, Sarah, V; Haslam, S. Alexander; Dingle, Genevieve A.; Jetten, Jolanda | GROUPS 4 HEALTH Reduces Loneliness and Social Anxiety in Adults With Psychological Distress: Findings From a Randomized Controlled Trial | unrelated topic | no technology | the intervention is not technological but a group activity |
| 2023 | Hernández-Ascanio, José; Perula-de Torres, Luis Ángel; Rich-Ruiz, Manuel; González-Santos, Josefa; Mielgo-Ayuso, Juan; González-Bernal, Jerónimo; Group, ASyS Study Collaborative | Effectiveness of a multicomponent intervention to reduce social isolation and loneliness in community-dwelling elders: A randomized clinical trial | insufficient data for pre-post correlation | no publically available data |  |
| 2024 | Hirshberg, Matthew J.; Dahl, Cortland J.; Bolt, Daniel; Davidson, Richard J.; Goldberg, Simon B. | Psychological Mediators of Reduced Distress: Preregistered Analyses From a Randomized Controlled Trial of a Smartphone-Based Well-Being Training | insufficient data for pre-post correlation | data are not publically available although OSF project exists |  |
| 2017 | Hulsbosch, Alexander M.; Nugter, M. Annet; Tamis, Petra; Kroon, Hans | Videoconferencing in a mental health service in The Netherlands: A randomized controlled trial on patient satisfaction and clinical outcomes for outpatients with severe mental illness | insufficient data for pre-post correlation | no publically available data |  |
| 2023 | Hussain, Z.; Ferreira, R.; Kuss, D.J. | The feasibility of smartphone interventions to decrease problematic use of social networking sites: A randomised controlled trial | insufficient data for pre-post correlation | no publically available data |  |
| 2022 | Chen, Yonghua; Liu, Xi; Chiu, Dorothy T.; Li, Ying; Mi, Baibing; Zhang, Yue; Ma, Lu; Yan, Hong | Problematic Social Media Use and Depressive Outcomes among College Students in China: Observational and Experimental Findings | unrelated topic | no technology | technology only as a complement to activities (group counseling consisted of two online activities and three offline activities) |
| 2022 | Christie, Hannah Liane; Dam, Alieske Elisabeth Henrike; van Boxtel, Martin; Köhler, Sebastian; Verhey, Frans; de Vugt, Marjolein Elisabeth | Lessons Learned From an Effectiveness Evaluation of Inlife, a Web-Based Social Support Intervention for Caregivers of People With Dementia: Randomized Controlled Trial | insufficient data for pre-post correlation | no publically available data |  |
| 2019 | Jarvis, M.A.; Padmanabhanunni, A.; Chipps, J. | An evaluation of a low-intensity cognitive behavioral therapy mhealth-supported intervention to reduce loneliness in older people | insufficient data for pre-post correlation | no publically available data |  |
| 2023 | Käll, A.; Andersson, G. | Knowledge acquisition following internet-based cognitive behavioural therapy for loneliness – A secondary analysis of a randomised controlled trial | results in another study |  | results already presented in another study |
| 2021 | Käll, A.; Bäck, M.; Welin, C.; Åman, H.; Bjerkander, R.; Wänman, M.; Lindegaard, T.; Berg, M.; Moche, H.; Shafran, R.; Andersson, G. | Therapist-Guided Internet-Based Treatments for Loneliness: A Randomized Controlled Three-Arm Trial Comparing Cognitive Behavioral Therapy and Interpersonal Psychotherapy | insufficient data for pre-post correlation | no publically available data |  |
| 2020 | Käll, Anton; Jägholm, Sofia; Hesser, Hugo; Andersson, Frida; Mathaldi, Aleksi; Norkvist, Beatrice Tiger; Shafran, Roz; Andersson, Gerhard | Internet-Based Cognitive Behavior Therapy for Loneliness: A Pilot Randomized Controlled Trial | insufficient data for pre-post correlation | no publically available data |  |
| 2021 | Kanter, Jonathan W.; Nash, Michael G.; Kuczynski, Adam; Rosen, Daniel C. | A BRIEF, MOBILE INTERVENTION TO DECREASE DEPRESSION AND LONELINESS AND IMPROVE RELATIONSHIP QUALITY DURING THE COVID-19 PANDEMIC | lack of required data |  | no pre and post data on loneliness in groups |
| 2022 | Kramer, Lean L.; van Velsen, Lex; Clark, Jenna L.; Mulder, Bob C.; de Vet, Emely | Use and Effect of Embodied Conversational Agents for Improving Eating Behavior and Decreasing Loneliness Among Community-Dwelling Older Adults: Randomized Controlled Trial | lack of required data |  | results are in the form of Δ M and SD |
| 2023 | Laakso, Mari; Fagerlund, Åse; Pesonen, Anu-Katriina; Figueiredo, Rejane A. O.; Eriksson, Johan G. | The Impact of the Positive Education Program Flourishing Students on Early Adolescents’ Daily Positive and Negative Emotions Using the Experience Sampling Method | unrelated topic | no technology | the intervention is not technological but well-being lessons |
| 2016 | Larsson, E.; Padyab, M.; Larsson-Lund, M.; Nilsson, I. | Effects of a social internet-based intervention programme for older adults: An explorative randomised crossover study | insufficient data for pre-post correlation | no publically available data |  |
| 2023 | Lim, Jung-Won; Park, Hwa-Ok Hannah; Kim, Min Jung | Effects of safety and care services on psychological outcomes and housing satisfaction in Korean middle-aged and older adults living alone | insufficient data for pre-post correlation | no publically available data |  |
| 2023 | Lin, Xin Yao; Zhang, Lin; Yoon, Saiyeon; Zhang, Ruoying; Lachman, Margie E | A Social Exergame Intervention to Promote Physical Activity, Social Support, and Well-Being in Family Caregivers | insufficient data for pre-post correlation | no publically available data |  |
| 2019 | Lindsay, E.K.; Young, S.; Brown, K.W.; Smyth, J.M.; Creswell, J.D. | Mindfulness training engages interpersonal processes: A randomized controlled trial | unrelated topic | no technology | all 3 groups had technology (audio lessons) |
| 2022 | Littlewood, Elizabeth; McMillan, Dean; Chew Graham, Carolyn; Bailey, Della; Gascoyne, Samantha; Sloane, Claire; Burke, Lauren; Coventry, Peter; Crosland, Suzanne; Fairhurst, Caroline; Henry, Andrew; Hewitt, Catherine; Baird, Kalpita; Ryde, Eloise; Shearsmith, Leanne; Traviss-Turner, Gemma; Woodhouse, Rebecca; Webster, Judith; Meader, Nick; Churchill, Rachel; Eddy, Elizabeth; Heron, Paul; Hicklin, Nisha; Shafran, Roz; Almeida, Osvaldo; Clegg, Andrew; Gentry, Tom; Hill, Andrew; Lovell, Karina; Dexter-Smith, Sarah; Ekers, David; Gilbody, Simon | Can we mitigate the psychological impacts of social isolation using behavioural activation? Long-term results of the UK BASIL urgent public health COVID-19 pilot randomised controlled trial and living systematic review | lack of required data |  | data in unadjusted/adjusted mean difference format |
| 2021 | Loveys, Kate; Sagar, Mark; Pickering, Isabella; Broadbent, Elizabeth | A Digital Human for Delivering a Remote Loneliness and Stress Intervention to At-Risk Younger and Older Adults During the COVID-19 Pandemic: Randomized Pilot Trial. | lack of required data |  | no pre and post data on loneliness in groups |
| 2021 | Loveys, Kate; Sagar, Mark; Zhang, Xueyuan; Fricchione, Gregory; Broadbent, Elizabeth | Effects of Emotional Expressiveness of a Female Digital Human on Loneliness, Stress, Perceived Support, and Closeness Across Genders: Randomized Controlled Trial | lack of required data |  | no pre and post data on loneliness in groups |
| 2024 | Lydon, Elizabeth A.; Mois, George; Shende, Shraddha A.; Myers, Dillon; Danilovich, Margaret K.; Rogers, Wendy A.; Mudar, Raksha A. | Methods and baseline characteristics for a social engagement technology-based randomized controlled trial for older adults | lack of required data |  | only baseline data |
| 2024 | Maj, Anna; Matynia, Maria; Michalak, Natalia; Bis, Aleksandra; Andersson, Gerhard | New in Town—An Internet-based self-efficacy intervention for internal migrants: A randomized controlled trial | insufficient data for pre-post correlation | data are not publically available although OSF project exists |  |
| 2018 | Mallow, Jennifer A.; Theeke, Laurie A.; Theeke, Elliott; Mallow, Brian K. | The effectiveness of mI SMART: A nurse practitioner led technology intervention for multiple chronic conditions in primary care | no RCT | experiment (other study design) | the effect of technology on, among other things, loneliness; but no RCT |
| 2023 | Mueller, N.E.; Cougle, J.R. | Building Closer Friendships in social anxiety disorder: A randomized control trial of an internet-based intervention | insufficient data for pre-post correlation | no publically available data |  |
| 2020 | Muralidharan, Anjana; Brown, Clayton H.; Zhang, Yilin; Niv, Noosha; Cohen, Amy N.; Kreyenbuhl, Julie; Oberman, Rebecca S.; Goldberg, Richard W.; Young, Alexander S. | Quality of life outcomes of web-based and in-person weight management for adults with serious mental illness | insufficient data for pre-post correlation | no publically available data |  |
| 2022 | Nooteboom, Peter | Social support across contexts: In times of crisis and in interventions | unrelated topic | no technology | both groups used technology |
| 2022 | Papadopoulos, Chris; Castro, Nina; Nigath, Abiha; Davidson, Rosemary; Faulkes, Nicholas; Menicatti, Roberto; Khaliq, Ali Abdul; Recchiuto, Carmine; Battistuzzi, Linda; Randhawa, Gurch; Merton, Len; Kanoria, Sanjeev; Chong, Nak-Young; Kamide, Hiroko; Hewson, David; Sgorbissa, Antonio | The CARESSES Randomised Controlled Trial: Exploring the Health-Related Impact of Culturally Competent Artificial Intelligence Embedded Into Socially Assistive Robots and Tested in Older Adult Care Homes | insufficient data for pre-post correlation | no publically available data |  |
| 2023 | Perkins, R.; Spiro, N.; Waddell, G. | Online songwriting reduces loneliness and postnatal depression and enhances social connectedness in women with young babies: randomised controlled trial | insufficient data for pre-post correlation |  |  |
| 2013 | Saulsberry, A.; Marko-Holguin, M.; Blomeke, K.; Hinkle, C.; Fogel, J.; Gladstone, T.; Bell, C.; Reinecke, M.; Corden, M.; Van Voorhees, B.W. | Randomized clinical trial of a primary care internet-based intervention to prevent adolescent depression: One-year outcomes | unrelated topic | no technology | no comparison of technology vs. no technology |
| 2024 | Seewer, N.; Skoko, A.; Käll, A.; Andersson, G.; Luhmann, M.; Berger, T.; Krieger, T. | Efficacy of an Internet-based self-help intervention with human guidance or automated messages to alleviate loneliness: a three-armed randomized controlled trial | insufficient data for pre-post correlation |  |  |
| 2021 | Shapira, Stav; Cohn-Schwartz, Ella; Yeshua-Katz, Daphna; Aharonson-Daniel, Limor; Clarfield, Avram Mark; Sarid, Orly | Teaching and Practicing Cognitive-Behavioral and Mindfulness Skills in a Web-Based Platform among Older Adults through the COVID-19 Pandemic: A Pilot Randomized Controlled Trial | insufficient data for pre-post correlation | no publically available data |  |
| 2013 | Shigaki, C.L.; Smarr, K.L.; Siva, C.; Ge, B.; Musser, D.; Johnson, R. | RAHelp: An online intervention for individuals with rheumatoid arthritis | lack of required data |  | loneliness results in M and SD only post-intervention + effect size |
| 2019 | Shorey, Shefaly; Chee, Cornelia Yin Ing; Ng, Esperanza Debby; Lau, Ying; Dennis, Cindy-Lee; Chan, Yiong Huak | Evaluation of a Technology-Based Peer-Support Intervention Program for Preventing Postnatal Depression (Part 1): Randomized Controlled Trial | lack of required data |  | no baseline loneliness data, only changes after 1 and 3 months based on a linear model |
| 2023 | Sun, L. | Social media usage and students' social anxiety, loneliness and well-being: does digital mindfulness-based intervention effectively work? | Retracted study |  |  |
| 2020 | Tsai, Hsiu-Hsin; Cheng, Ching-Yu; Shieh, Wann-Yun; Chang, Yue-Cune | Effects of a smartphone-based videoconferencing program for older nursing home residents on depression, loneliness, and quality of life: a quasi-experimental study | insufficient data for pre-post correlation | no publically available data |  |
| 2021 | Valeri, Linda; Amsalem, Doron; Jankowski, Samantha; Susser, Ezra; Dixon, Lisa | Effectiveness of a Video-Based Intervention on Reducing Perceptions of Fear, Loneliness, and Public Stigma Related to COVID-19: A Randomized Controlled Trial | lack of required data |  | loneliness data only at baseline and only N |
| 2011 | Weinert, C.; Cudney, S.; Comstock, B.; Bansal, A. | Computer intervention impact on psychosocial adaptation of rural women with chronic conditions | insufficient data for pre-post correlation | no publically available data |  |
| 2019 | Westerhof, Gerben J.; Lamers, Sanne M. A.; Postel, Marloes G.; Bohlmeijer, Ernst T. | Online Therapy for Depressive Symptoms: An Evaluation of Counselor-Led and Peer-Supported Life Review Therapy | insufficient data for pre-post correlation | no publically available data |  |
| 2002 | White, H; McConnell, E; Clipp, E; Branch, LG; Sloane, R; Pieper, C; Box, TL | A randomized controlled trial of the psychosocial impact of providing internet training and access to older adults | lack of required data |  | data in medians (interquartile range) |
| 2022 | Zengin Alpozgen, A.; Kardes, K.; Acikbas, E.; Demirhan, F.; Sagir, K.; Avcil, E. | The effectiveness of synchronous tele-exercise to maintain the physical fitness, quality of life, and mood of older people - a randomized and controlled study | lack of required data |  | results are in the form of Δ M and SD |
