# Supplementary figures and images for: Efficacy of Technology-Based Interventions on the Reduction of Loneliness: Systematic Review and Meta-Analysis
Source: J Med Internet Res. 2026 May 8;28:e80059. doi: 10.2196/80059 (PMC13158556; doi:10.2196/80059)

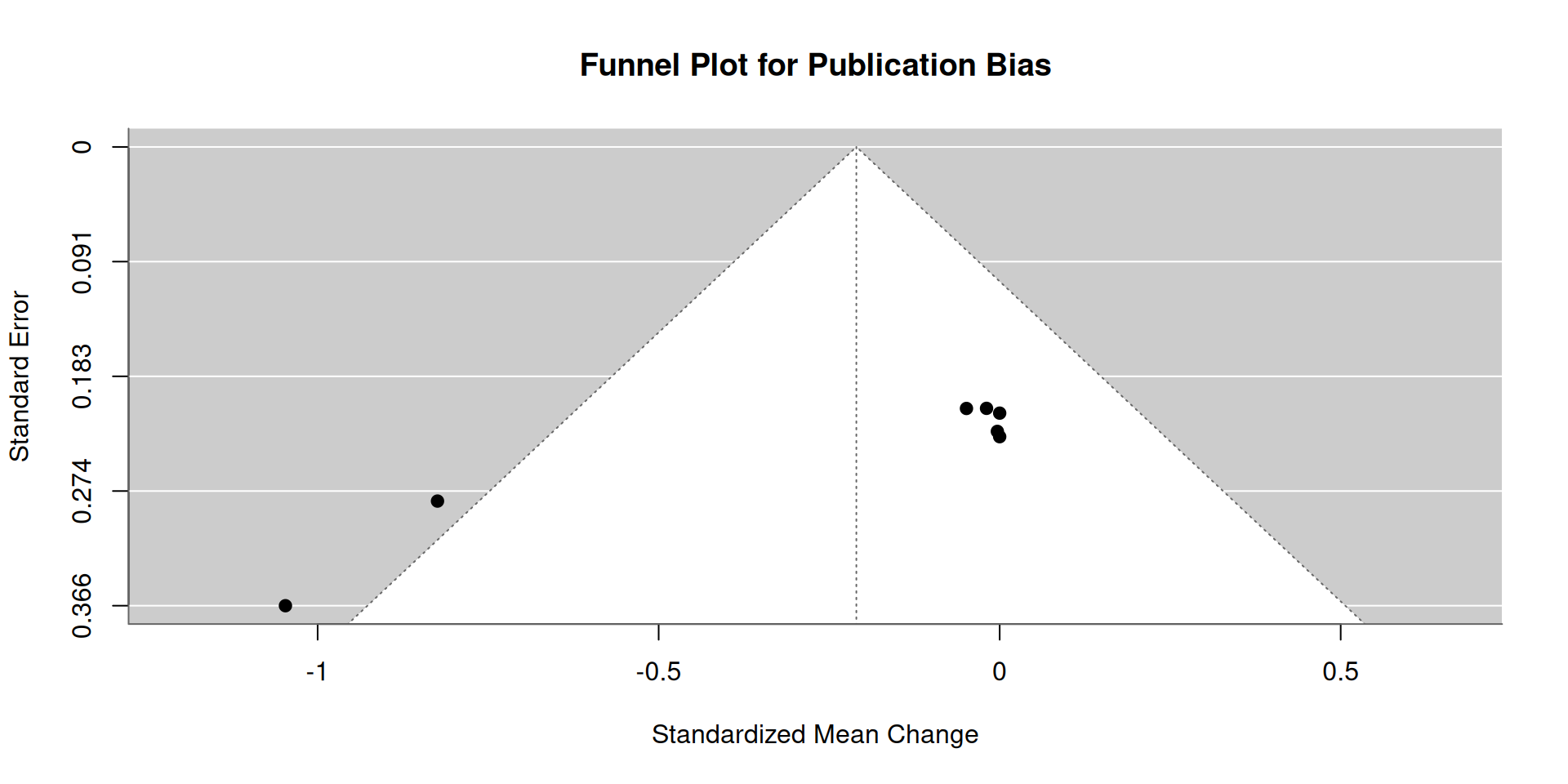

Supplement: Multimedia Appendix 3 [file jmir-v28-e80059-s003.png]
