# Supplementary material for: Efficacy of Technology-Based Interventions on the Reduction of Loneliness: Systematic Review and Meta-Analysis
Source: J Med Internet Res. 2026 May 8;28:e80059. doi: 10.2196/80059 (PMC13158556; doi:10.2196/80059)
Supplement: Multimedia Appendix 4 [file jmir-v28-e80059-s004.docx]

**Multimedia Appendix 4**

**Table S1.**GRADE summary of findings: technology-based interventions compared to control conditions for reducing loneliness.^a^

| **Certainty assessment** | | | | | | | **Number of participants** | | **Effect** | **Certainty** | **Importance** |
| --- | --- | --- | --- | --- | --- | --- | --- | --- | --- | --- | --- |
| **N studies** | **Study design** | **Risk of bias** | **Inconsistency** | **Indirectness** | **Imprecision** | **Other considerations** | **Technology-based interventions** | **Control conditions (wait-list, usual care, no intervention)** | **Absolute (95% CI)** |  |  |
| 7 | Randomized trials | Serious^b^ | Serious^c^ | Not serious^d^ | Serious^e^ | None | 301 | 279 | SMD^f^ –0.21 (–0.59 to 0.17) | ⨁◯◯◯ Very low^a,b,c,d^ | CRITICAL |

**Notes:** ^a^Loneliness assessed with University of California, Los Angeles Loneliness Scale.^b^The majority of included studies (5 out of 7) were assessed as having either ’some concerns’ or ‘high risk of bias.’ Specifically, two studies were rated as high risk, primarily due to issues with missing outcome data and deviations from intended interventions. Three studies raised some concerns, mainly related to the lack of participant blinding, which is inherent to psychosocial interventions.^c^Downgraded by one level due to moderate heterogeneity (P=.033) and a wide prediction interval (–1.14 to 0.63), which indicates that the true effect in future studies could range from a substantial reduction in loneliness to a potential increase.^d^The evidence directly answers the review question. The included studies evaluated the target interventions (technology-based) in the relevant populations and measured the primary outcome of interest directly using a validated scale (University of California, Los Angeles Loneliness Scale).^e^The 95% CI (–0.59 to 0.17) crosses the null effect line (zero) and encompasses both the possibility of a moderate reduction in loneliness and a small increase.^f^SMD: standardized mean difference.
